# Connectivity and Order: an Analytical Framework

**DOI:** 10.1007/s12140-023-09401-z

**Published:** 2023-02-21

**Authors:** Bart Gaens, Ville Sinkkonen, Henri Vogt

**Affiliations:** 1 Finnish Institute of International Affairs (FIIA), Helsinki, Finland; 2 University of Turku, Turku, Finland

**Keywords:** International order, Regionalism, Connectivity, Indo-Pacific

## Abstract

IR literature has become inundated with different descriptions for the future of international order. The coming age is purportedly marked by China’s ascendancy, American decline, a leaderless “no-one’s world”, or multiple competing modernities. Yet the global fight against climate change or shared COVID-19 strategies convey a different image of the world’s predicament. The situation appears paradoxical: increasingly tense great-power relations are mixed with ever-strengthening interdependencies. This article contributes to these debates by exploring how global orders as well as regionalism today are increasingly defined by various types of connective functional links between intentional actors at various levels of social organisation. To enable a nuanced analysis, the article introduces an analytical framework composed of six *connectivity* logics, namely cooperation, copying, cushioning, contestation, containment, and coercion. These play out differently within material, economic, institutional, knowledge, people-to-people, and security spheres. The utility of this article’s approach is demonstrated through empirical examples related to the policies of key actors in the Indo-Pacific region.

## Introduction

Today’s world is marked, paradoxically, by both deepening interconnectedness at all levels of society and intensifying attempts to disrupt connections that are deemed unsavoury, whether these be in the form of information networks, financial flows, or even people. Taking a cue from this, our article—and this special issue—seeks to clarify the contours of the concept of *connectivity*, linking it to key debates on international order and regionalism in the process.

While connectivity is much discussed in current policy parlance—particularly in the context of different “connectivity strategies” pursued, e.g. by China and the European Union—the term is rarely defined with sufficient precision and risks becoming a mere empty signifier. To rectify this, we unpack connectivity into an analytical framework composed of, first, six distinguishable *connectivity spheres:* infrastructural, economic and financial exchange, institutional, knowledge exchange, societal action, and security. These constitute the material and human domains of interaction within which the other dimension of our framework operates, namely, six different *logics of connectivity:* cooperation, copying, cushioning, contestation, containment, and coercion. Through the employment of these logics, we wish to underline how connectivity requires some degree of (strategic) intentionality on the part of the actors engaged in the processes of connection or disconnection. *Agency* thus appears central to the production of (dis)connectivity, although connectivity pursuits may produce unintended consequences in the form of positive or negative externalities.

In addition to the benefits of conceptual clarification, the sense of nuance offered by our framework appears important from a *normative* point of view. We believe that a densely networked world in which the major (state) actors, and most smaller ones too, seek to create functional connections with other actors, particularly with those in their vicinity, on a single-issue basis within a range of different spheres and by relying on inclusive and constructive connective logics, would prove relatively stable but still dynamic. It would be a world in which unavoidable mutual confrontations hardly coalesce into major power-political crises, a world in which spheres of influence necessarily remain blurred. We thus adhere to a form of pragmatic, functional, intentional rationality as a guide for state action, to what we call *pragmatic connectivism*. Under such rationality assumptions, the world would appear by no means anarchic but rather thickly ordered; instead of focussing on survival, states would seek to navigate as cleverly as possible in these orderly waters.

The article sets out by situating our argumentation within broader international relations debates on polarities, global ordering, and the idea of regions and region-building. This overview is vital in view of the observation that connectivity is a key component of today’s global order and various efforts of constructing regionalised spheres of influence. The second section engages in a theoretical and conceptual discussion of connectivity in light of the nascent body of literature on the notion. The final and most important section introduces the two-dimensional analytical framework of connectivity by way of empirical examples derived from the Indo-Pacific region—a fluid regional constellation where different types of (dis)connectivity seem to materialise in a particularly distinct manner. The master narrative framed by these examples shows how different (state) actors intentionally assume different logics and roles within a large, dynamic, functional entity, thus seeking to serve the interests of their citizenries (or elites?) in one way or the other.

## From Global Polarities to Regional Constellations

In recent years, already before Russia’s criminal war against Ukraine, it has been in vogue to argue that the international order is in a state of flux, with concomitant implications for the regional make-up of the globe. While there may be some semblance of agreement that a change indeed is afoot, there is no consensus on the relevant parameters for describing, let alone understanding, said transformation.

At the most basic level, much ink has been spilled debating the possible shifts in the *polarity* of the international system, measured predominantly in terms of military and economic power capabilities. Some scholars point to the resilience of America’s unipolar position or a variation thereof. Brooks and Wohlforth ([1]: 68–71), for example, foresee America’s lead in material terms persisting well into the twenty-first century, bringing about a “1+y+x” world, defined by a US lead in capabilities, followed by a China that again stands apart from the other great powers of the day. For others, like Maher [2], the world is entering a period of “rough” or “loose” bipolarity, distinguishable from the Cold War version by the profound interdependence—economic and otherwise—between the two poles of China and the USA. Yet others posit that the impending global system will be multipolar, owing to the rise of new great powers (especially China but also others like India and Russia) and the “relative economic decline” of the USA and its allies (e.g. [3]). In this reading, a multipolar world defined by competing power centres and potentially shifting alliances will be a far more dangerous and unpredictable place than either the brief unipolar post-Cold War interregnum or the bipolar system of the Cold War.

However, it is legitimate to question how useful it is to think of the international system in terms of polarities and balance of power. Polarity-focussed accounts miss the inherent social nature of power relations, downplay the role of actors that do not belong to the select group of great powers, and ignore domestic politics ([4]: 23–26; see also [5]). Other variables, like the nature of relations between actors (i.e. whether or not they are interdependent), as well as domestic political factors, like quality of leadership or national resolve, invariably need to be brought into the analysis. For us, then, polarity is at best a material background condition for a much more interesting concept that captures trappings of stability beyond capability-based indicators—namely *order*.

In his work, Acharya [6] makes an important distinction between *descriptive* and *normative* understandings of the concept. In the first case, order is simply equated with the “status quo” or “an existing distribution of power or an institutional arrangement, irrespective of its consequences for peace or conflict”. In the latter instance, order “stresses some desirable objectives, such as increased stability, predictability, if not peace per se in international relations” ([6]: 5). This second understanding draws from Bull’s ([7]: 8) famous conception of international order as “a pattern of activity that sustains the elementary or primary goals of the society of states”. Orders, thus understood, have a function and they serve a (higher) purpose, to enable states to maintain sovereignty and provide public goods or at minimum to escape from the prospect of disorder or chaos [8]. Order guarantees a sense of freedom of action, of agency; it involves a level of “purposive [social] construction” ([9]: 854–855).

Thus, conceptualised, international order is made up not only of rules and values that define the parameters of permissible action—what Cooley and Nexon [10] have termed its “architecture”—but also the various *interactions and connections* between actors, the order’s “infrastructure”. Institutional fora, then, are the sites where architecture and infrastructure meet, where crucial interactions between key actors take place, and where rules of the road are agreed upon and compromises become possible.

Ikenberry [8] argues that international order can be built around three different ideal-typical mechanisms (cf. “logics” in our vocabulary): the balance of power, command, and consent. They rely on disparate “sources of authority” such as sovereignty, material power, and rule of law; have different “moral purposes”, i.e., preservation of autonomy, interests of the dominant state, and provision of public goods; and produce varying constellations of hierarchy, e.g. co-equal great powers, rulers/subjects, and leaders/followers. However, each of these ordering mechanisms lends a special role for powerful actors as creators and managers of order, although this is less pronounced in consent-based orders. The much-discussed liberal international order is no exception. The USA has always acted as that order’s manager and operator, “not just the sponsor and leading participant” ([8]: 193).

Critics have been quick to point out, however, that the reach of this liberal order, as well as America’s pursuits in building an order with genuinely consensual characteristics, remained predominantly confined to the “West”. Hence, when the liberal order finally reached momentary global supremacy after the end of the Cold War, the seeds of its eventual fragmentation were laid by expansion. Kupchan [11] has termed this new global constellation a “no one’s world” of contending power blocs espousing potentially irreconcilable ideologies. Maintaining order in such a world requires willingness to tolerate more diverse governance arrangements, in a word, pluralism. For Acharya [4], this world resembles a “multiplex”, a world without a global hegemon, with many kinds of actors linked through *complex connections* and fragmented governance frameworks. Here different modernities coexist in “cultural, ideological and political diversity, including alternative pathways to stability, peace and prosperity” ([4]: 16). Flockhart [12], similarly, conceptualises a multi-order world, where various international societies with smaller memberships are embedded in an international system and agency becomes more diffuse than in the past, existing both above and below the state, regionally and supranationally. The analytical framework of connectivity introduced in the present article broadly subscribes to these views.

One prominent way to fathom the world of the twenty-first century, then, is in terms of different *regions and concomitant regional orders*. Employing regions as units of analysis and policy-making gained in prominence especially during the post-Cold War era (see, e.g. [13, 14]), as a result of a more permissive American stance towards regionalism, increasing global interdependence, and new positive attitudes towards neoliberal dogmas in the developing countries. The evolution of regional order-building has, however, by no means been a linear phenomenon but, as many scholars have pointed out, rather a matter of several distinct phases: e.g. the trade-oriented regionalism of the 1950s and 1960s; the regionalism of the 1980s and early 1990s based on (neo)liberal political principles and conditionalities on the part of such actors as the European Union; and the interregionalism and transregionalism of the late 1990s and 2000s, involving a great range of civil society and business actors [15–18].

In the 2010s, the contours of regional dynamics have clearly changed again. Two mutually related factors have shaped this (ongoing) transformation. First, the relative importance of interregional cooperation has been declining. As a result of weakening multilateralism, increasing multipolarity and great-power competition, the EU, for example, has started to emphasise “complex interregionalism” [19], aiming to establish differentiated interregional relationships with a much stronger role given to bilateral relations and minilateral arrangements. Second, and concomitantly, various intentional logics of connectivity have increasingly come to shape the world’s (regional) orders; owing to the diversity of the connective logics, these orders remain fluid in nature, with geographical boundaries imprecisely defined. This process has been driven forward, above all, by China’s efforts to create spheres of interest and influence in Asia as well as in Europe and Africa. Other actors have responded, seeking to balance China and construct connectivity partnerships in other regions, thus expanding their respective spheres of influence.

This in fact conjures up the notion of “superregion”, developed *inter alia* in the context of regional security complexes by Buzan and Waever [13]. Ideas such as Atlanticism, the Asia-Pacific, or pan-Americanism represent superregional arrangements based on economic integration as well as mutual defence and security arrangements; in all of these, the USA appears to be an actual member. But Buzan and Waever also argued that the rise of China might herald the beginning of a nascent Asian supercomplex, a set of regional security complexes (RSCs), “within which the presence of one or more great powers generates relatively high and consistent levels of interregional security dynamics” ([13]: 492).

More recently, Rory Medcalf ([20]: 473) has utilised the concept of superregion to depict the current *Indo-Pacific* constellation, “a super-region with hard-to-define outer limits and distinct subregions yet an unquestionably Asian core” with “outer boundaries [that remain] fluid”. The emergence and evolution of this superregion is marked by the growing centrality of the region’s powerful actors in security, economic, institutional, and diplomatic terms. As a result, the Indo-Pacific space is now permeated with a myriad of connective tissues, “the arc of trade routes, energy flows, diplomatic bonds and strategic connections between the two oceans”. These regional dynamics are intimately tied to China’s growing connectedness with other players in its vicinity; the notion of the Indo-Pacific therefore does not *only* represent an exclusionary device for containing and/or shutting out Beijing ([20]: 471–472; see also [21]). Indeed, postulating that connectivity plays an increasingly salient role in today’s region-making as well as policy-making, and not only in the Indo-Pacific, warrants a more systematic look into the concept and its ramifications.

## Connectivity and Its Conceptual Underpinnings

Connectivity is still an academically underdeveloped concept—in spite of the fact that recent research has discussed it, as Sommerer and Tallberg [22] note, against the backdrop of such different factors as geography, cultural legacies, market mechanisms, and institutional arenas. It even seems that in empirical milieus, the concept has prompted more comprehensive efforts of systematisation.

To our knowledge, the most useful real-life definition of connectivity originates from the Asia-Europe Meeting (ASEM), a multilateral forum for dialogue and cooperation between 51 states from Asia and Europe, also including the European Union and the ASEAN Secretariat. The forum reached a consensus on the definition in 2017 and stipulated that, in general, connectivity is about bringing countries, people, and societies closer together. The concept covers hard connectivity (infrastructure projects) but also soft connectivity (people-to-people or digital connectivity), and all links: land, sea, air, cyber, and educational connections, as well customs cooperation and trade facilitation. ASEM also agreed that connectivity has to be in line with international standards and based on full transparency and that sustainability needs to be a quality benchmark, including the implementation of the Sustainable Development Goals [23, 24]. Building on this definition, Ries [25] has defined connectivity as “all the ways in which states, organisations (commercial or else) and societies are connected to each other and interact across the globe”, including physical flows, information flows, “hard” infrastructures, and “soft” regulatory measures or socio-cultural ties.

This wide-ranging definition implies that connectivity has ramifications in multiple dimensions. Connectivity is key to many traditional processes of regional integration, such as, for example, in the Association of Southeast Asian Nations (ASEAN), an organisation that focuses strongly on connections and infrastructure development while avoiding the strong political undertone of EU-style integration. Moreover, connectivity carries a number of political, security, or development-related connotations. The building of infrastructures, for example, is often heavily securitised, imbued with geopolitics, and linked to development cooperation. China’s investments in Central Asia or in Europe as part of its Belt and Road Initiative have important political and security implications. Development assistance, an important form of connectivity, has become increasingly securitised and has turned into a key component of the geostrategic use of economic power in many regions of the world [26]. A strong element of competition thus often underlies connectivity, as key actors aim to establish contending spheres of interest, for example, through infrastructure development. At times we can even speak of “connectivity wars” that play out through (geo)economic warfare, the weaponisation of international institutions, and infrastructure competition [27].

As for other scholarly efforts to make sense of connectivity, Parag Khanna’s is possibly the best known. Khanna, in his *Connectography* [28], shamelessly celebrates this current era of connectivity: “Like liberty or capitalism, [connectivity] is a world-historical *idea*, one that gestates, spreads, and transforms over a long timescale and brings about epochal changes. [...] Connectivity is thus more than a tool; it is an *impulse* ([28]: 7; emphasis in original).” Khanna’s understanding of connectivity is primarily based on material *infrastructures*, on highways, pipelines, and cables. It is geographical in the *functional* rather than political sense, which means, among other things, that the policies and role of major cities will centrally determine future global constellations and that previous hostilities are bound to turn, in the name of functional rationality, into cooperation. In our view, however, the constraining factors of strategic thinking that all state and non-state actors habitually undertake seem to figure to too limited a degree in this flow of optimism—as if a *deus ex machina* ultimately produced beneficial linkages between actors. The underlying impetus of connectivity remains un(der)defined.

The above insights—in particular, the idea-based functional focus of connectivity coupled with potential security implications and linkages to developmental efforts—are highly useful for our two-dimensional connectivity framework presented in the next section. In addition to them, four other aspects in (current) connectivity (strategies) deserve special emphasis. First of all, connectivity always involves an element of *intentionality*, of active agency, although this is not necessarily articulated in relevant policy documents. In this respect, it is actually a straightforward and even transparent phenomenon. This contrasts, for example, to the much subtler logic of Foucauldian *gouvernementalité*, currently widely utilised in analyses of global governance (e.g. [29]). Connectivity does *not*, a priori, require any piecemeal (discursive or identity-political) transformation on the part of the actor to be connected, and if it does, the connector-to-be usually explicitly articulates the conditions for required change. Indeed, self-regulation, usually through law, is an important dimension of (dis)connectivity strategies (cf. the article by Mattlin and Rajavuori in this Special Issue).

Second, the various forms of intentional connectivity generally involve an element of *imagined futures* [30]. Established connections function, to an extent, in the same manner as economic investments that are to produce concrete profits for the investor sometime in the foreseeable future. Moreover, the future-orientation of connections enables novelty, the emergence of economic and governance innovations, and new and profitable ways of doing things in various fields of life; the ultimate nature of each individual connection is not predetermined. The connections are thus closely attached to the capitalist mode of exchange and interaction in the world. This does not, of course, downplay the role of the past in connectivity politics; along with geographical proximity, historically evolved cultural similarities are indeed the most common precondition for connectivity.

Third, connectivity is a continuous, *constantly changing* process that can be consensual or contentious; the relative stability of traditional alliance building or of pole systems is simply not there. By extension, the space of manoeuvre and the patterns of *agency* of state, civic, and business actors alike are under constant need of recalibration, creating a *dynamic order* within which various logics of action constantly emerge and simultaneously operate. By way of example, smaller states such as Singapore or Finland are continuously forced to assess the nature of their strategic choices and the utility of their normative and connective frameworks often dominated by more powerful actors. Concomitantly, the regulatory and monitoring capabilities of non-state actors continuously depend on the willingness of global and regional powers to “open up” the space of governance. One example is the regulation of telecommunications where the USA, and to a lesser extent the EU, has primarily relied on private actors, whereas China has attempted to maintain regulatory powers within the International Telecommunications Union (ITU) in order to create a state-led system of internet regulation.

Lastly, connectivity goes hand in hand with *disconnectivity*. Countries impede economic transactions by way of tariffs and even sanctions, with the aim to disconnect. Donald Trump’s “America First” policy towards European countries, with a strong economic component involved, appeared to be, in essence, a matter of disconnectivity. Historically, in the real-socialist Europe of the Cold War, citizen rights movements consciously tried to establish links with their Western counterparts, seeking to connect their countries with the West and disconnect them from the Soviet hemisphere. Preferences of consumption patterns are also noteworthy in this context—even individuals possess (dis)connective agency. When one refrains from buying a Huawei phone in Europe for political reasons, this may be seen as an attempt of micro-level disconnection.

## Spheres and Logics of Connectivity

Let us now bring forth our two-dimensional analytical framework of connectivity, aspiring to both grasp the complexity of the phenomenon and providing a conceptual toolkit for further analyses, including the other articles in this Special Issue. The framework consists of six *spheres* (or fields of connectivity) and six *logics* (different ways of connecting). It covers the activities and policy articulations not only of state actors but also transnational and multinational corporations, big and small, as well as sub-state actors such as nongovernmental organisations, individual citizens, and consumers (see Table 1 on page 9). As indicated above, key to this actorness is strategic intent; perceived strategic preferences determine actors’ willingness to connect and disconnect [31]. These interests can relate to, for example, economic growth, supply chain efficiency, or resilience in terms of diversified connectivity to counteract supply chain disruptions ([32]: 3–5).

**Table 1:** Spheres and logics of connectivity with examples drawn from the Indo-Pacific

**Spheres of connectivity**	**Logics of connectivity-disconnectivity**					
	***Cooperation:*** *creation of inclusive mutually beneficial connective networks*	***Copying:*** *enhancing connectability by emulating others*	***Cushioning:*** *broadening the range of options through connectivity to mitigate risks*	***Contestation:*** *gaining advantage(s) over other actors through connectivity*	***Containment:*** *partial or complete exclusion of an actor through disconnecting or by building exclusive zones of connectivity*	***Coercion: f****orcing others to connect a certain way or forgo certain connections altogether*
*Infrastructural*	Infrastructure partnerships (e.g. Asia Africa Growth Corridor, AAGC)	Japan’s CAREC as model for BRI	Sri Lanka cushioning against China-India rivalry	Japan’s Partnership for Quality Infrastructure (PQI) posing viable alternative to China’s controversial BRI; Japanese and Chinese EEZs in Myanmar	Android updates in Huawei devices stopped by the USA	Disruptions in Eurasia Rail connections by transit countries seeking higher transit fees
*Economic and financial exchange*	Trade facilitation; India produces the West’s vaccines	Japan’s “economic cooperation” as model for China’s ODA	Pakistan between EU and China; India’s energy supply diversification after Iran sanctions; India and China decreasing dependency on USD	China Pak Economic Corridor (CPEK) as geoeconomic tool to contest India’s predominance in South Asia	(CP)TPP excluding China	China’s rare earth sanctions against Japan (2010)
*Institutional frameworks of governance*	COVAX; alignment with Paris climate change agreement	Japan changing its privacy law to echo EU GDPR; FOIP construct adopted by Japan’s partners	India in Quad and RIC or SCO	China’s shadow institutions (AIIB, SCO, Shanghai GFC...) contesting Western institutions	Quad excluding/containing China	South China Sea Arbitration under UNCLOS (2016)
*Knowledge exchange*	BioNTech-Pfizer (US-EU); EU-ASEAN energy cooperation	ASEAN Power Grid emulating EU-Mediterranean electricity ring	Alternative cyber routes (e.g. Arctic Connect)	Competing 5G technologies (Huawei, Nokia, Ericsson)	Limitations to Chinese students in the USA; China’s “cyber sovereignty”	Digital disruption; election meddling (e.g. China-Taiwan)
*Socio-cultural*	EU support to higher education in the ASEAN region (SHARE)	Bollywood copying Hollywood; Hong Kong democracy movement	Cities cushioning against negative effects of urbanisation by promoting inclusive tourism	Contending narratives on the Comfort Women issue between South Korea and Japan	Visa regimes; limitations to tourism	Forced migration, eviction, resettlement (e.g. Southeast Asia)
*Security*	Anti-piracy operations (ATALANTA); BRI 2.0 in Myanmar	UK, France, Germany FONOPS in the South China Sea approximating US operations	India participating in Quad and conducting military exercises with Russia	Chinese and Japanese naval bases in Djibouti	Regular military exercises (e.g. MALABAR)	ADIZ in East China Sea; Extraterritorial repression; China’s flash military exercises around Taiwan

To illustrate the usability of the framework, we apply the Indo-Pacific as a case study of sorts. In current policy parlance, the notion of the Indo-Pacific primarily figures as a strategic and security-grounded concept. It constitutes a functional “rescaling” of Asian regionalism, from mere Asia-Pacific economic integration to an Indo-Pacific security-focussed agenda [33]. On the surface, this process seems to be driven forward by the quadrilateral response by the USA, Japan, India, and Australia to China’s increasing maritime assertiveness, naval modernisation, SLOCs, and territorial claims. The present article, however, shows that the picture is much more convoluted. The Indo-Pacific epitomises “a world of flows” [34], marked by numerous variable geographies driven forward by connectivity in all its dimensions and in a wide array of fields. If we collect the empirical viewpoints exemplifying each of the six logics below (i.e., the italicised paragraphs below), an illustrative master narrative of these complex dynamics emerges.

### Connectivity Spheres

We call the material and human fields of interaction through and in the context of which intentional connectivity materialises *spheres,* and distinguish between six of them.^1^ These categories are, in many instances, *overlapping and interlinked*—unavoidably given the all-encompassing nature of the framework—but they do have an independent core within which the different logics of action, the other dimension, can materialise.

• The most elementary of these six spheres covers all *material infrastructures* that establish concrete connections and in fact exclude those who cannot share, or are not part of, these infrastructures. These include energy and transport networks, e.g. aviation and train connections and the corresponding *regulations* of these, and digital infrastructures that make the flow of information, ideas, and capital possible.

• All kinds of *economic transactions* compose the second sphere in our conceptualisation. Many or even most of the (existing) transactional practices in this sphere do not initially involve any intentionally connective element, but once the economic linkage between two actors is there, it is likely to generate conscious policies and practices that seek to guarantee the feasibility of such transactions in the future.

• The third sphere concerns the *institutional frameworks of governance* and the concomitant norm and rule production of the world. These frameworks can be global or regional in nature; they can be highly specific or fairly general, and they can even be regime constellations within a field. We include, for example, investment and trade treaties under this category or the numerous failed attempts to reach trade treaties. Ultimately, the question is of interpretations and reformulations of international law—which can be seen as an institutional framework in itself.

• As for the fourth sphere, *knowledge exchange* plays a hugely important role in the current world, as the successes of developing COVID-19 vaccines have shown. Research diplomacy, i.e. cooperation in R&D, sharing expertise and exchanging data and information, clearly belongs to this sphere.

• The fifth sphere covers all kinds of *socio-cultural* exchange across the region. In this people-to-people context, the level of intention in terms of connectivity may sometimes appear minimal, but when we, for example, think of diaspora populations across the world—say, the Chinese in the neighbouring countries—they often have openly expressed agendas to support their country of origin*.*^2^

• The final sphere of our framework is that of *security*, in many respects also an overarching theme, one that possibly underlies all the other efforts to establish connectivity within a region and in world politics more generally. This category naturally encompasses a plethora of activities, from joint operations to patrol the high seas through traditional alliance building all the way to using hybrid tools to influence political decision-making in other countries.

### Connectivity Logics

Along with these connectivity spheres, we distinguish between six possible *logics* underlying connectivity. As is the case with the spheres, there are real-world overlaps in these logics, but they do, in our view, capture the most essential ways through which this multifaceted phenomenon currently materialises. Two important binaries in terms of the consequences of these logics are ever present: *inclusivity versus exclusivity*, namely the degree to which each logic opens or closes connective spaces, connects rather than disconnects; and *constructiveness versus corrosiveness*, i.e. the extent to which the logics breed a more or less sustainable order through connections. Moreover, it is clear that some of these logics are more prominent on the level of state action than others, but it is beyond the scope of this article to assess the level of relative prominence of each of the logics; in any case, this would be a highly demanding empirical endeavour. It is also important to note that vantage points are important: since connectivity is by definition relational, an action or policy might appear informed by one logic from the standpoint of one actor and another logic from the standpoint of a second one. The logics are discussed in order from positive to negative below.

The first logic of connectivity—namely *cooperation*—refers to intentional actors engaging in connective endeavours as a means to obtain mutual benefits, although these benefits need not necessarily accrue equally to all the connected actors. Such forays can encompass various constellations; they can be bi-, tri-, mini-, pluri-, or multilateral or even multi-stakeholder in nature; often the mere coordination of policies to serve some commonly agreed-upon end state meets the criteria of cooperation. The onus is thus on enabling or *empowering*, exercising power *with* as opposed to power *over* others, and in the process creating inclusive connected networks. Cooperative connectivity can also produce positive cycles of connectivity relations, where increased interactions and transactions between actors allow them to engage in social learning, “an active process of redefinition or reinterpretation of reality” ([38]: 43). This process can over time allow actors to establish new regional identities around shared norms and even “security communities” defined by expectations of peaceful change.

Cooperative connective relationships thus require actors to possess some degree of *trust* in their counterparts. At minimum, actors must harbour a “belief that one will not be harmed when one’s interests are placed in the hands of others” ([39]: 246) in order for the connective relationship to endure. On a deeper level, of course, trust can also be founded upon normative expectations that the other party will do what is morally upstanding or right in a particular normative framework.

*The plethora of Indo-Pacific infrastructure initiatives by key players such as China, the USA, Japan, and the EU, and also by multilateral development banks and regulatory dialogue fora, provide opportunities for building cooperative, synergetic linkages in terms of capital, knowledge/expertise, and dialogue/capacity-building* [40]*. Coordination within cooperative institutions on international standards, principles, and procedures is a key first step in this regard. As another example, the growing strategic rivalry between the USA and China has driven ASEAN and the EU, strategic partners since December 2020, closer together. Free trade agreement negotiations are back on the table, and the EU has strongly supported the ASEAN Customs Transit System. Moreover, a dialogue to share best practices on energy security and diversification of the energy mix including renewables, in line with the EU-ASEAN Plan of Action (2018–2022), is in the works.*

*In the field of governance, we see an ever-expanding web of FTAs, including the Regional Comprehensive Economic Partnership (RCEP), which has restored hopes in reviving free trade negotiations in a trilateral China-Japan-Korea FTA, or the Comprehensive and Progressive Agreement for Trans-Pacific Partnership (CPTPP). Knowledge exchange is another good example, as in the case of EU-Japan cooperation in peaceful civilian use of space* [41]*. Countries also seek to entice sharp “foreign” minds to their universities; the US has successfully employed this strategy for decades but other big states, such as Australia and China, have consciously increased their corresponding efforts in this field in recent years, at least regionally.*

We term our second logic *copying.* There are two sides to this. On the one hand, countries or actors that feel themselves inferior in some respect—or somehow “inconnectible”—can seek to copy, say, institutional structures, governance mechanisms, or best practices of another actor and thus improve their chances of future connections. In IR literature, such dynamics are captured through terms like contagion [42], attraction [43], the power of example [44], and emulation [45]. While all of these describe diffuse social processes wherein some practice, norm, or value is deemed as inherently worthy of imitation, it pays to stress that the “copier” is not a passive receiver but an active participant, even if the line between coercion (see below) and emulation might at times appear blurry. Copying can also be done in a clandestine manner to achieve some useful end. Chinese attempts at industrial espionage in the West to gain access to military-technological know-how are a case in point.

On the other hand, actors possessing something worth copying need not necessarily actively promote those assets. Others can be attracted, for instance, to a vibrant economy, a democratic system of government or a particular business model even if these are not advertised. As Nye ([43]: 92) has argued, certain attributes of an actor can enhance its attractiveness in the eyes of others through appearing benign, competent, or even charismatic. Being copied, then, implies that an actor possesses “soft power”. This form of power is co-optive, draws on attributes like values, culture, and upstanding conduct, and is contingent on the willingness of others to be attracted or persuaded, as opposed to compelled [43].

*The proliferation of the entire Indo-Pacific construct, and especially the adoption/adaptation of the originally Japanese Free and Open Indo-Pacific (FOIP) concept in the policy parlance of the USA, Australia, and India, constitutes an example of copying an entire intellectual frame for looking at the regional constellation. China’s model of infrastructure investment in other countries can be seen as including elements of copying. For example, China has arguably emulated the Asian Development Bank’s CAREC programme: the Central Asia Regional Economic Cooperation. Started in 1997 and with a leadership role by Japan, CAREC proposed six economic cooperation corridors, reminiscent of China’s economic belts across Eurasia as part of the BRI (*[46]*: 12* [47]*: 463). China has also “copied” Japan’s model of economic cooperation and development assistance* [26]*. Since the 1970s, Japan provided assistance through economic cooperation to developing countries—China was one of them at the time—mainly consisting of large official loans for natural resource development, with the aim to secure the imports of raw materials to Japan. In the same vein, China has tried to safeguard its own economic interests through couching its foreign aid as a component of broader economic cooperation. Both countries have thus taken their own national interest and recipient countries’ self-help as starting points for assistance, and focus on building long-term economic and political partnerships, often with middle-income countries (MICs).*

Our third logic of *cushioning* in international relations implies that an actor—usually a small, middle-size, or regional power but also others like an IO, a corporation, or even a city—can position itself between two (or sometimes more) major actors and in a sense get the best out of both of these worlds. The Finnish policies during the Cold War represent a useful case in point: the country maintained good relations both with the Soviet Union and the Western world and could utilise this position to raise its relative weight in world politics and for its own economic benefit. In IR literature, this approach is often captured under the banner of hedging. Actors seek to control risk in dangerous or volatile environments by making limited or diversified commitments to two or more other actors and also by making decisions that might at first blush appear to work in opposite directions ([48]: 333 [49]: 761). Cushioning could feasibly be practiced by actors both “above” and “below” the state (the literature on hedging in IR remains state-centric) and take place vis-à-vis actors that straddle these various levels of analysis. It need not, then, be confined to actions taken towards two or more great powers, even if it often is.^3^

*Hedging policies by smaller Indo-Pacific states can be regarded as examples of cushioning. Against the background of increasing Chinese interest and investment in places such as Colombo and Hambantota Port, and India as the neighbourhood’s predominant power, Sri Lanka has utilised the rivalry between both these major powers but avoided confrontation with them. In its counter-terrorism efforts, for example, Sri Lanka has been cushioning vis-à-vis both China and India, enticing Beijing to offer material and diplomatic support, while convincing New Delhi to offer intelligence, non-lethal weapons, and economic assistance* [51]*.*

*Contestation* refers to our fourth logic of connectivity. Here, actors are trying, more or less fiercely, to gain advantages over others within specific spheres of connectivity, *without* the express intention of shutting out those with whom they engage in such competitive endeavours. This broad understanding of contestation covers actors competing in or over markets, geographical spaces (whether on land, at sea, or in aerospace), and the production of knowledge, as well as construction of and wrangling over institutions and norms. In this normative sphere, contestation entails “a social practice of [...] objecting to norms (principles, rules, or values) by rejecting them or refusing to implement them, and as a mode of critique through critical engagement in a discourse about them” ([52]: 109). In institutional settings, contestation can include both “voice” and “exit” [53], although in the latter case these institutions would need to be, at least in principle, relatively open to participation, lest they turn into instruments of exclusion. Similarly, clashing narratives over the meaning and even naming of connective spaces—the term “Indo-Pacific” being a prime example—fall under the remit of contestation. Contestation, moreover, is often rule-regulated; the idea is to create alternatives and gain an advantage within a mutually accepted (but possibly contested) framework of norms and practices. Contestation thus unfolds through pursuits in connecting, not disconnecting.

*Contestation often occurs simultaneously with cooperation. The security threat inherent in so-called dual-use serves as a representative example: investments in infrastructure or technology can have links with military ends. Scientific diplomacy in the realm of satellite tracking or connectivity initiatives such as underwater data cables can raise concerns about dual-use capabilities. As regards the latter, China’s participation in the Arctic Connect underwater data cable project connecting Europe with Russia and Asia along the Northern Sea Route may pose a security threat, as this could justify Chinese military presence in the Arctic to protect its strategic infrastructure* [54]*. China has also been creating parallel structures and alternative institutions to increase its global influence, contestatively but without strongly questioning the existing international order (*[55]*: 89). China’s shadow institutions include the Conference on Interaction and Confidence-Building Measures in Asia (CICA) as an alternative to the ASEAN Regional Forum (ARF), the AIIB as a substitute for the ADB, and the Shanghai Global Financial Centre (GFC) to contend with London and New York* [56]*. These can be seen as “important [first] steps to shape the ecology of international order” in China’s preferred direction (*[10]*: 104).*

Our fifth logic, *containment*, entails actors endeavouring to exclude, to disconnect, another actor or group of actors, or considerably circumscribe its or their ability to act within a particular sphere—and at the same time enhance connectivity with others. Containment thus encompasses an *exclusionary* logic vis-à-vis another actor or groups of actors, an element not found in contestation, and it can be both constructive and corrosive of order by creating deeper connective links within an in-group while seeking to undermine an out-group. In traditional structural realist terms, containment has a strong “external balancing” dimension.^4^ Dueck ([58]: 31) argues that as a strategy, containment is “defensive” because it seeks “to prevent expansion, deter, and deny hostile gains” and works through “balancing, drawing lines, and creating geopolitical counterweights”.

It pays to stress, however, that containment is not merely a military strategy. There has been a revived focus on *economic* containment in recent years [59], particularly with the rise of “decoupling” rhetoric in the West and the idea of “dual circulation” in China [60, 61]. *Diplomatic* containment of adversaries and pariahs has been a common practice, whether one looks at Saddam Hussein’s Iraq, post-revolution Iran, the military junta in Myanmar, or, in the 1990s and currently, the Taliban in Afghanistan. Therefore, although containment is almost habitually associated with great-power strategies, other actors (or collections of actors) can feasibly practice it, although it is manifestly more difficult if the target of containment is sufficiently more powerful than the “container(s)” and thus able to counter or circumvent containment efforts.

*China’s infrastructure investment under the banner of the BRI has led to a range of contestative and/or containing responses by other actors. Japan, for example, has successfully promoted its Quality Infrastructure concept in the international community as an alternative to the controversial BRI. Since 2013 Tokyo has thereby acquired an advantageous position in the international infrastructure market as a capable provider and credible partner in minilateral arrangements (*[46]*: 17). Any traditional effort of military alliance building of course belongs to this sphere, whatever the underlying justification of that effort (e.g.* [62]*), as well as minilateral, issue-based cooperation in the security sphere at the tri- and quadrilateral level. The Australia–United Kingdom–United States Partnership (AUKUS) is a recent high-profile example. Similarly, the Quad, in spite of its informal and low-key character, can also be seen as a countermove, aiming to contain China in one way or the other. The group’s profile has risen recently, with four hitherto unprecedented leader-level summits held in 2021 and 2022. In the summits, the group has pledged to, for instance, strengthen the Paris Agreement on climate change, to amp up efforts to provide COVID-19 vaccines to states in the Indo-Pacific region and promote the building of high-standard infrastructures* [63]*.*

The final logic of connectivity that we wish to bring to the fore is *coercion*. In short, it entails “the infliction of pain or damage – or the withdrawal of something valued, such as patronage or affection – to compel an actor to behave in a particular way” ([64]: 58), that is, to connect or to disconnect. Here we thus associate coercion primarily with a Dahlian conceptualisation of power, without making aprioristic assumptions about the means through which it is exercised. Coercion can occur through a multitude of instruments, whether these be military, economic, institutional, diplomatic, or symbolic in nature ([65]: 49–50). When it comes to coercive connectivity, it is obvious that this logic has played a prominent role throughout history, particularly in imperial pursuits. The forced opening up of the Chinese market by Western powers in the nineteenth century is an instructive case in point, one whose historical reverberations are felt to this day. A caveat worth noting is that coercion often appears a destructive logic when it comes to constructing and maintaining a sustainable regional *order* (in the normative sense of the term, at least). Coercion is a potentially high cost and normatively dubious way to achieve connectivity; it is, in effect, corrosive of legitimacy and the social bases of order.

*The recent flash military exercises undertaken by China’s People’s Liberation Army (PLA) around Taiwan constitute an obvious yet topical exercise of “coercive diplomacy”* ([43]: 39–48)*. As clear examples of weaponised connectivity/interdependence, actors have utilised forced migration, including exploiting and manipulating outflows created by others (see also *[66]*: 78). The suppression of the Rohingya in Myanmar has resulted in mass-scale forced migratory movements to Bangladesh. Furthermore, disregard of local stakeholders in Special Economic Zones (SEZ) resulting in forced evictions and resettlements can be seen as tools for coercion. The China-run Dawei and Japan-operated Thilawa SEZs in Myanmar are cases in point. Lastly, extraterritorial repression by authoritarian states, ranging from obscuring negative news about their governments to silencing activists and political opponents in third countries, can be seen as bluntly revealing “the dark side of connectivity”* [67] *in terms of coercion.*

## Conclusion

This article has argued that in order to understand the ongoing shifts in global order dynamics, it is useful to think of the world in terms of geographically undetermined regional constructs that are increasingly shaped by various forms of connectivity. The paragraphs above have highlighted how the connective endeavours materialise in six distinct spheres and are rooted in six different logics. These can be cross-tabulated into a table of 36 cells, manifesting the diversity and overarching nature of the phenomenon—as epitomised by the Indo-Pacific regional space. In policy-relevant terms, if connectivity tends more towards the left-hand side of the table above, towards cooperation, then we can expect a more stable, peaceful, and less anarchical order. If it leans towards the right side, towards coercion, the opposite is the case. The idea(l) of pragmatic connectivity, the article’s underlying normative proposition, entails harnessing the full mix of connective strategies while being mindful of the downsides of exclusionary practices, particularly containment and coercion—they should only be employed as a last resort. Indeed, actors subscribing to this ideal in their policies strongly emphasise the potential *future* benefits of the connective networks.

Three tentative conclusions can be drawn at this stage. First, moving beyond debates on system polarities and global ordering, our focus on connectivity reveals how different forms of power can be exercised with, as well as against, other actors through the establishment, management, and severing of connective relationships. Power, in this framework, is an inherently *relational* and *situational* concept rather than a reflection of capabilities or a notion that is limited to the mere ability to preclude other actors from pursuing certain policy courses.

Second, through the various forms of connectivity, actors, whether states, international organisations, cities, or corporations, can create a multitude of connections to serve distinct *functional* ends. They may, for instance, seek to maintain systemic stability, integrate their actions with those of others in a new manner, control a certain policy area, or seek to have a bigger say in it in the future. This is rooted in the idea that international order is a purposively constructed entity that actors on different levels of social organisation seek to shape through intentional agency.

Third, the constructed and connected order in which said actors participate has *transformative* potential vis-à-vis those very actors. Put differently, connections are not only made by actors; actors are also made by connectivity. This (re)constitution of actors’ interests and identities materialises through the variegated processes of connecting and through the long-term employment of these connections as a means of partaking in the order. Herein resides the transformative potential of employing constructive and inclusive connective logics. A connected order defined by consensual and absolute-gains-based connections should, in the long run, predispose its constituent actors to prefer such approaches as the basis of interaction with others, rendering the order more stable, less exploitative, and more normatively desirable.

In sum, it is the nature, interplay, and totality of different connections that make regional orders dynamic and alive. Studying connections across micro- and macro-levels provides novel lenses for fathoming the mechanisms of regional order maintenance, management, erosion, and even transformation.

## Footnotes

1 It goes without saying that this is just one possible way of categorisation. Johnston’s [35] framework on different orders and China’s support for them is one example. Furthermore, in the field of globalisation studies, Appadurai [36] has introduced five dimensions or “scapes” that underlie global cultural flows: ethnoscapes, technoscapes, financescapes, mediascapes, and ideoscapes.

2 It is noteworthy that sociologists currently pay a great deal of attention to what they call connected (or even connective) histories (and, by extension, connectivist sociology). In order to understand the contours of (current) modernity, we need to bring in histories from the “margins” and connect them with what is usually perceived as “mainstream” [37].

3 It also pays to note that great powers, especially regional ones, can also practice cushioning vis-à-vis two global superpowers ([50]: 138).

4 The notion is prominently discussed in IR literature on US Cold War grand strategy [57], but the remit of containment is broader, and actors have practiced it since time immemorial.
